# A regulatory SNP modifies cystic fibrosis by disrupting NFκB complex binding on FAS

**DOI:** 10.1186/2194-7791-2-S1-A2

**Published:** 2015-07-01

**Authors:** Chidiebere U Awah, Silke Hedtfeld, Burkhard Tümmler, Frauke Stanke

**Affiliations:** Department of Paediatrics Pneumology and Allergology, Hannover Medical School, Biomedical Research in Endstage and Obstructive Lung Disease Hannover(BREATH), Member of the German Center for Lung Research, Hannover, Germany; Molecular Medicine, Hannover Biomedical Research School, MHH, Hannover, Germany

## Meeting abstract

Cystic Fibrosis (CF) is the most common autosomal recessive disease affecting about 1 in 3200 in Caucasians with increased morbidity, chronic infections, disability and reduced life expectancy. The mutations causing CF are well characterized, but the gene-gene and gene-environment interactions which significantly modify CF disease severity are completely unknown.

We report the pathogenetic mechanisms underlying how a regulatory SNP modifies Cystic Fibrosis (CF). Association studies of affected individuals with Cystic Fibrosis identified the causal variant, a regulatory SNP rs7910656 on the non-coding region of *FAS* gene on human chromosome 10q24.1, which includes the binding site of many transcription factors especially the NFκB complex, but no molecular alterations were detected by conventional approaches. Using a combination of functional in-silico gene expression analysis and computational transcription factor binding analysis together with electrophoretic mobility shift and Supershift assays, we have identified that an allele of the regulatory SNP disrupts the binding of the master transcription factor NFκB on *FAS* and which likely interferes with normal immune activation and programmed cell death.

Thus, our work demonstrates for the first time how a regulatory SNP disrupts the binding of a master transcription factor NFκB and a key signaling pathway FAS in immune regulation and programmed cell death modifying Cystic Fibrosis.

